# Thermal Performance of Novel Multilayer Cool Coatings for Asphalt Pavements

**DOI:** 10.3390/ma12121903

**Published:** 2019-06-13

**Authors:** Yujing Chen, Kui Hu, Shihao Cao

**Affiliations:** 1 College of Civil Engineering and Architecture, Henan University of Technology, Zhengzhou 450001, China; yujingchen@gmail.com (Y.C.); emailshc@163.com (S.C.); 2 National Engineering Laboratory for Wheat & Corn Further Processing, Henan University of Technology, Zhengzhou 450001, China

**Keywords:** asphalt pavement, cool coating, coating structure, cooling effect, temperature field

## Abstract

Cool coatings are typically used to address high-temperature problems with asphalt pavements, such as rutting. However, research on the effect of the coating structure on the cooling performance remains a major challenge. In this paper, we used a three-layer cool coating (TLCC) to experimentally investigate the effects of the reflective layer, the emissive layer, and the thermal insulation layer on the cooling effect using a self-developed cooling effect evaluation device (CEED). Based on the test results, we further established temperature fields inside uncoated and coated samples, which were used to study how the TLCC affects the inner temperature field. Our results showed that the reflective layer was the main parameter influencing the cooling effect (8.18 °C), while the other two layers were secondary factors that further improved the cooling effect to 13.25 °C. A comparison of the temperature fields showed that the TLCC could effectively change the internal temperature field compared with the uncoated sample, for example, by reducing the maximum temperature inside, whose corresponding position was also deeper. As the depth increased, the cooling effect of the TLCC first increased and then decreased slowly. The results emphasize the importance of considering the effect of the coating structure on the cooling performance. This study provides a reference for effectively alleviating high-temperature distresses on asphalt pavement, which is conducive to the sustainable development of pavements.

## 1. Introduction

Asphalt pavements have the advantages of high flatness, riding comfort, and low noise and have been widely used in engineering construction. However, asphalt mixtures have a high absorption rate for sunlight, especially in summer, which causes the temperature of the asphalt pavement to rise sharply. On the one hand, high temperature is not good for the asphalt pavement itself. For example, high temperature tends to cause the asphalt mixture to soften easily, and with the continuous rolling of vehicles, it may produce viscous flow and even road distress, such as rutting [1,2]. This greatly increases the maintenance cost and reduces the life of asphalt pavement, which is detrimental to the sustainable development of asphalt pavement. On the other hand, high-temperature asphalt pavements can exacerbate the urban heat island (UHI) effect, thereby accelerating global warming [3,4].

Cool coatings are considered an effective technical means because of passive cooling and resistance to the UHI effect. Initially, cool coatings were popular on roofs as a relatively inexpensive technology used to reduce buildings’ energy requirements for cooling during the summer [5]. In recent years, the application of cool coatings on asphalt pavements has attracted increasing attention. At present, its application on roads mainly focuses on the following aspects:

1. Development of high-performance functional fillers [6,7,8] and pigments, especially nonwhite pigments with high infrared reflectivity [9,10,11], such as Bi^3+^-doped and Bi^3+^/Tb^3+^-co-doped LaYO_3_ pigments [12]. Further, Xie et al. noted that coatings doped with chrome are a feasible way to improve the reflectance of sunlight, especially near-infrared reflectivity [13].
2. Durability of the coating. Peng et al. [14] investigated the hydrophobic and anti-icing performance of silicone coating. Li et al. [15] researched the life cycle assessment of reflective coatings for pavements.
3. Impact on the UHI [16,17,18,19]. Qin et al. [20] suggested that raising the albedo of pavements could effectively improve the urban canyon albedo only when the canyon has a low aspect ratio (e.g., h/w ≤ 1).

However, research on coating structures is still in its infancy. Although cool coatings for asphalt pavements have achieved good cooling performance, most coatings are single-layer structures. This means that some functional materials do not perform their intended function. For example, some reflective functional materials may be wrapped by other opaque materials, thereby weakening their optical reflection function. Therefore, the single layer structure limits the future development of the cool coating. In other words, it is still a considerable challenge to study the effect of the structure on the cooling performance. Moreover, another important issue is that there are few studies that focus on how cool coatings affect the temperature field inside the asphalt pavement, which is important for the softening of asphalt mixtures [21].

The purpose of this article is to prepare and test the cooling effect of a coating containing three structural layers, that is, the TLCC (three-layer cool coating). First, we developed a cooling effect evaluation device (CEED) and methods to ensure the accuracy of the test results. Second, we tested the effect of each structural layer on the cooling effect of the coating through the above device and determined the content of key materials in each layer. Finally, the influence of the coating on the internal temperature field of the whole sample was analyzed. This paper contributes to alleviating the problems caused by high temperatures, such as rutting.

## 2. Design Principles of Cool Coatings

**Figure 1:**
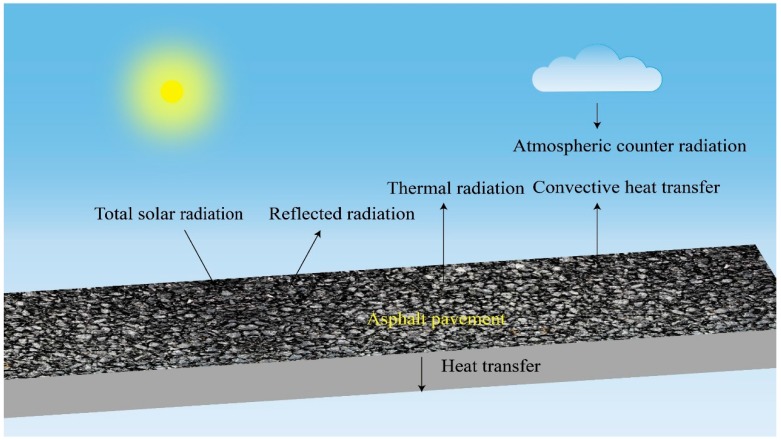
Thermal environment of asphalt pavement.

Asphalt pavement is in a complex light and heat environment. Figure 1 shows that asphalt pavement can absorb, reflect, and emit radiation and that it can also transmit it downward by conduction and upward by convection. The above factors can be quantitatively described by Equation (1). When the asphalt pavement is in thermal equilibrium, its calculation formula is as shown in Equation (2).

(1) $$q^{\prime }=\alpha \left(G_{S}+G_{sky}\right)-G_{sur}-G_{h}$$

where:

$q^{\prime }$―specific rate of heat flow, W/m^2^;
α―absorptivity of total solar radiation;
$G_{S}$―total solar radiation, W/m^2^;
$G_{sky}$―atmospheric counter radiation, W/m^2^;
$G_{sur}$―thermal radiation, W/m^2^;
$G_{h}$―convective heat transfer, W/m^2^.

When $q^{\prime }=0$,

(2) $$\alpha \left(G_{S}+G_{sky}\right)=G_{sur}+G_{h}$$

If the heat inside the asphalt pavement is reduced, we can control the following aspects: increase the reflected radiation (α) and increase the emitted thermal radiation ($G_{sur}$). In addition to the above, it is also necessary to consider reducing the heat transfer downward.

**Figure 2:**
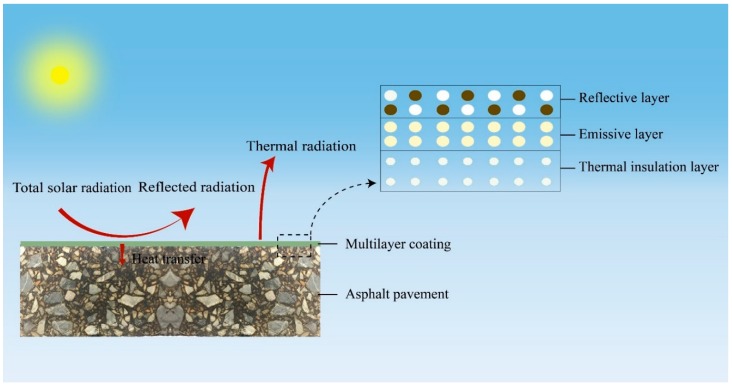
Working principle of the three-layer cool coating (TLCC).

Therefore, we have proposed a design of the TLCC (three-layer cool coating), the structure of which is shown in Figure 2. The design of the cool coating is as follows: (1) The first layer, the reflective layer, whose main purpose is to reflect visible light and infrared light, ensures that as little light as possible enters the coating and passes down, while the layer’s surface glare should be considered. (2) The second layer, also called the emissive layer, is mainly intended to emit radiation. The emissive layer pumps energy into the surrounding space by thermal radiation, which is a passive cooling method that is important for reducing the temperature of the object and mitigating the urban heat island effect. (3) The third layer, the thermal insulation layer, is responsible for preventing heat from being transferred downward into the interior of the asphalt pavement.

## 3. Materials and Methods

### 3.1. Materials

#### 3.1.1. Cool Coatings

According to the design principle of the TLCC with multifunctional layers, key functional materials consist of reflective materials, radiant materials and insulating materials. We chose rutile titanium dioxide [22] as the reflective material, silicon dioxide [23,24] as the radiant material, and hollow glass microbeads as the insulating material to study the influencing factors of the cooling performance of the TLCC, as shown in Table 1, Table 2, and Table 3. Then, epoxy resin was chosen as the adhesive material with high transparency. Furthermore, to form a high-performance coating, some other additives were used, for example, a diluent agent, curing agent, dispersing agent, and antifoaming agent. Finally, carbon black of different proportions was selected to alleviate driving glare.

#### 3.1.2. Asphalt and Mixtures

Basalt was chosen as the aggregate, and the gradation of the asphalt mixture was AC-16 type. The optimum content of asphalt binder is 4.6 wt %, which was obtained from the Marshall test. The preparation process of the asphalt mixture conformed to China’s specification of JTG F40-2004. The size of the rutting board made of the asphalt mixture was 300 mm × 300 mm × 50 mm.

### 3.2. Methods

#### 3.2.1. Preparation of Test Samples

**Figure 3:**
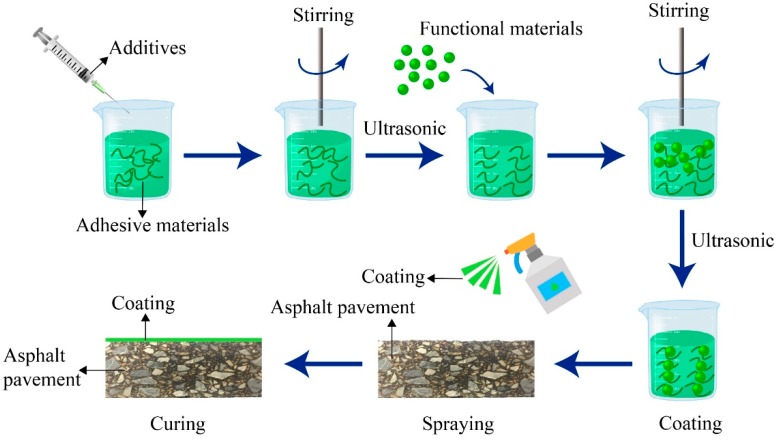
Schematic of the coating preparation process.

To spray coatings evenly on the standard rutting board, an ultrasonic dispersing instrument and stirrer were used for the operation. As shown in Figure 3, the specific preparation process was as follows:

1. Modifying the bonding material. To improve the properties of the adhesive material (epoxy resin), it was necessary to use additives, such as dispersing agents and antifoaming agents.
2. Adding functional materials. Although the functional materials of each layer of the cool coating were different, their operation steps were the same.
3. Spray coating. Coating was applied to the surface of the rutting plate. Each coating layer was 0.3 kg/m^2^.
4. Curing. The curing speed is related to the ambient temperature, and the higher the ambient temperature, the faster the curing speed. In this test, the temperature condition of 40 °C was selected.

Based on the above steps, we investigated the effect of each functional layer on the overall cooling performance of the cool coatings by the control group and different test groups, as shown in Table 4.

#### 3.2.2. Cooling Effect Test

**Figure 4:**
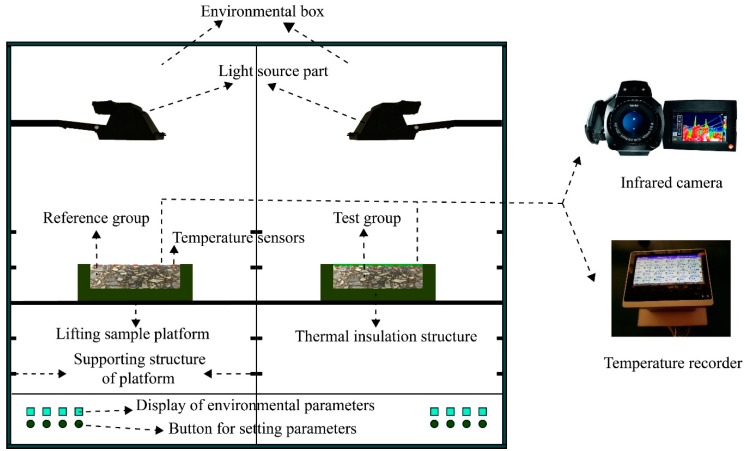
Self-developed cooling effect evaluation device (CEED).

We designed a test device (CEED) to evaluate the cooling performance of cool coatings, as shown in Figure 4. To ensure the accuracy of the test results, the device mainly had the following design features:

1. First, the device had to be capable of controlling environmental parameters and eliminating the effects of different environmental factors on measurement accuracy, such as air temperature and humidity.
2. Then, to simulate sunlight, we used tungsten halogen lamps, whose spectrum is similar to that of sunlight. We adjusted the angle of incidence to emit parallel light, which was achieved by a suitable lampshade design.
3. Furthermore, to best simulate the actual solar radiation in different weather conditions, we adjusted the intensity of the light striking the surface of the road simulation structure (the sample).
4. Finally, to ensure the accuracy of the test dates, we took measures, such as wrapping the sides and bottom of samples with a 5 cm-thick layer of thermal insulation materials, to reduce the heat loss during the test.

**Figure 5:**
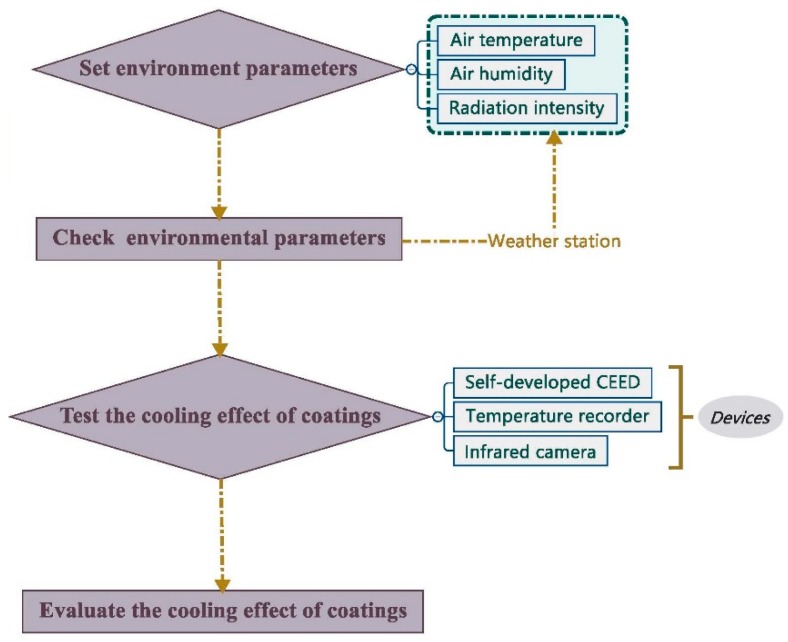
Specific steps of the cooling effect test.

In addition to the indoor CEED, temperature recorders, infrared cameras, weather stations and other devices were applied in this test. These devices worked together to complete the cooling effect test work according to certain process steps. As shown in Figure 5, the specific steps of our test were as follows:

1. The working environment parameters were set, including air temperature, humidity, and radiation intensity, where the temperature was 20 °C, the humidity was 50% RH, and the equivalent radiation intensity was 700 W/m^2^.
2. The environmental parameters were checked. At the preparatory stage prior to the test, the weather station helped check and determine environmental parameters, such as radiant intensity.
3. The cooling effect of the coatings was tested. When the light source was turned on, the sample entered the heating stage. The temperature data were collected using a temperature recorder and temperature sensors every 12 minutes. After the samples reached thermal equilibrium, the thermal infrared images were taken on the side of the samples using an infrared camera, followed by temperature field analysis.
4. The cooling effect of the coatings was evaluated. The temperature difference between the coated and uncoated samples was calculated using Equation (3), where ΔT is used to indicate the cooling performance of the coating:

(3) $$\Delta T={\mathrm{Temperature}}_{uncoated}-Temperature_{coated}$$

## 4. Results and Discussion

### 4.1. Reflective Layer

#### 4.1.1. Content of Titanium Dioxide

**Figure 6:**
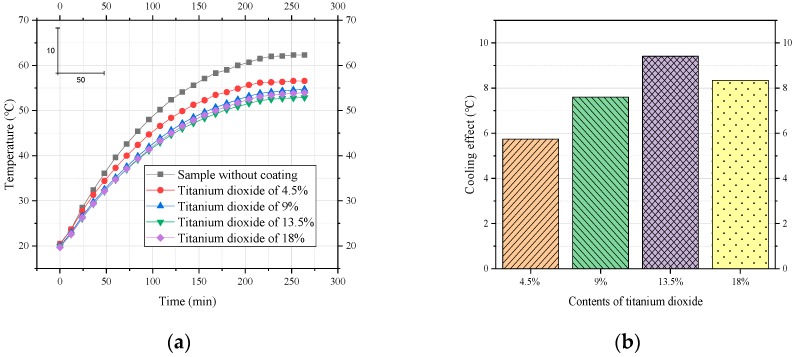
Effect of titanium dioxide on the cooling performance of coatings. (**a**) Process of heating to thermal equilibrium; (**b**) comparison of cooling effects.

To evaluate the titanium dioxide content in the reflective layer, the doses of titanium dioxide were selected to be 4.5%, 9%, 13.5%, and 18%. The time histories of rising temperature are shown in Figure 6a, and the comparative cooling effects are shown in Figure 6b.

As Figure 6 shows, the cooling effect at first increased with an increase in the content of titanium dioxide. When the percentage of titanium dioxide changed from 4.5% to 13.5%, the cooling effect significantly increased, reaching 9.41 °C. Titanium dioxide is a kind of material with a high refractive index and wide band gap that can reflect most visible light and near-infrared light. Therefore, with the increase in titanium dioxide particles, the cooling performance of the coating was improved. However, when the content of the reflective functional material reached 18%, the cooling effect of the coating did not increase but decreased slightly. This may be due to the aggregation of some particles, which resulted in a reduction in the specific surface area of heat dissipation and thus a reduction in scattering efficiency. Therefore, we recommend 13.5% titanium dioxide in the reflective layer.

#### 4.1.2. Carbon Black in the Reflective Layer

To study the effect of carbon black content on the cooling performance of the coating, its proportion was chosen as the only variable at 0.4%, 0.6%, and 0.8%. Other ingredients and their proportions were the same.

**Figure 7:**
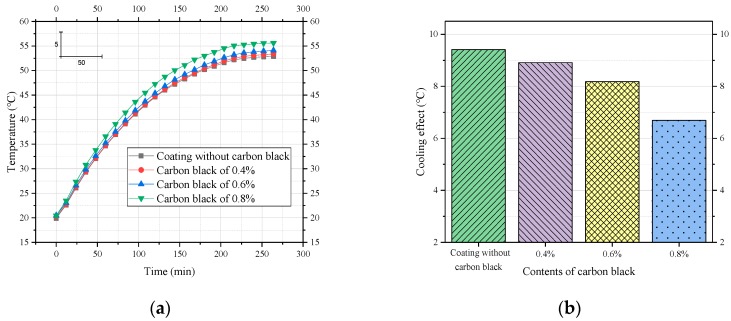
Effect of carbon black on the cooling performance of coatings. (**a**) Process of heating to thermal equilibrium; (**b**) comparison of cooling effects.

Figure 7 shows that the use of carbon black reduced the cooling effect of the coating, and as the amount of carbon black increased, the cooling effect of the coating became lower. The use of carbon black (0.4%) caused a slight decrease in the cooling effect of the coating, from 9.41 °C to 8.91 °C, a decrease of 0.5 °C. When the amount of carbon black was increased from 0.4% to 0.6%, the change in cooling effect was still small, decreasing by 0.73 °C; however, when the dose of carbon black was increased to 0.8%, the cooling effect of the coating was significantly decreased, a drop of 1.49 °C. This trend suggests that carbon black had an adverse effect on the cooling effect of the coating while improving surface glare. Moreover, large doses of carbon black resulted in increased costs. Taking these factors into consideration, we recommend a carbon black content of 0.6%.

### 4.2. Emissive Layer

A single-layer coating of silicon dioxide is the simplest and most promising coating for enhancing the emissive layer. To determine the effect of the emissive layer on the overall cooling performance of the coating, a coated sample with an emissive layer was used as a test group, while a coated sample without an emissive layer was used as a reference group. Furthermore, to determine the content of silicon dioxide in the emissive layer, its percentage was chosen to be 13%, 18%, and 21%.

**Figure 8:**
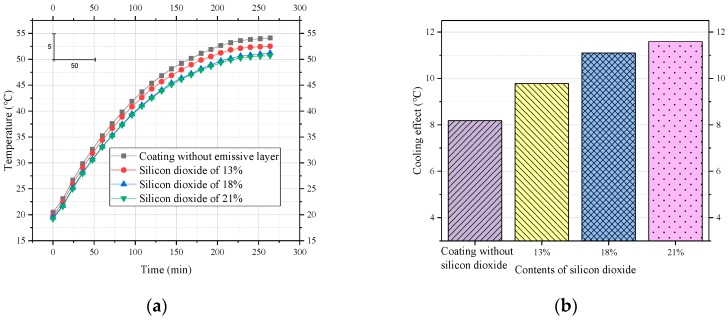
Effect of silicon dioxide on the cooling performance of coatings. (**a**) Process of heating to thermal equilibrium; (**b**) comparison of cooling effects.

Figure 8 shows that the emissive layer had a positive effect on the cooling performance of the coating. In particular, the application of the emissive layer allowed the coating to have a cooling effect of more than 8.18 °C. As the amount of silicon dioxide increased from 13% to 18%, the cooling effect of the coating increased from 9.78 °C to 11.1 °C, an increase of 1.32 °C. When the amount of silicon dioxide was increased to 21%, the increase in the cooling effect of the coating was small, only 0.49 °C. It should be noted that as the filler (silicon dioxide) increased, the viscosity of the coating increased. However, the viscosity must be controlled within a suitable range to maintain its workability. Therefore, it was necessary to control the content of silicon dioxide. Therefore, we recommend a silicon dioxide content of 18%.

### 4.3. Thermal Insulation Layer

**Figure 9:**
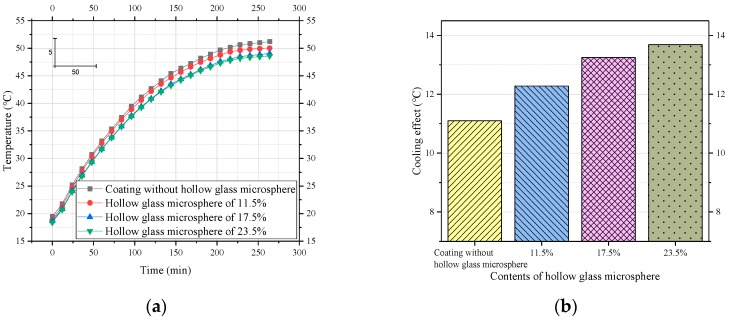
Effect of hollow glass microbeads on the cooling performance of coatings. (**a**) Process of heating to thermal equilibrium; (**b**) comparison of cooling effects.

The coating layer without the insulating layer was used as a reference group, and 11.5%, 17.5%, and 23.5% of hollow glass microspheres were added to prepare a test group. As shown in Figure 9, the coating with the thermal insulation layer had a better cooling effect. Specifically, the cooling effect of the coating exceeded 11.1 °C due to the thermal insulation layer. A content of 11.5% of the hollow glass beads increased the cooling effect of the coating by 1.18 °C, from 11.1 °C to 12.28 °C. When the content of the hollow glass microspheres continued to increase to 17.5%, the cooling effect was still satisfactory, increasing by 0.97 °C. Since hollow glass beads have a hollow structure and low thermal conductivity, they can improve the thermal insulation performance of the coating. However, when its content was increased to 23.5%, the cooling effect of the coating did not increase much, increasing only by 0.44 °C. Moreover, as the content of hollow glass microspheres increased, the compressive modulus and strength of the composites decreased. Therefore, the use of an appropriate amount of microspheres allows the coating to have a satisfactory overall performance. We recommend using 17.5% hollow glass beads in the thermal insulation layer.

### 4.4. Temperature Fields of the Sample Inside

**Figure 10:**
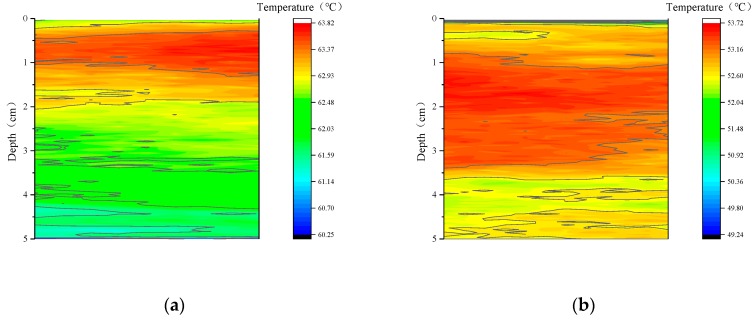
Temperature fields of samples inside an (**a**) uncoated sample and a (**b**) coated sample.

To investigate the cooling effect of the coating inside the sample, the coated (TLCC) and the uncoated rutting board were taken as the research samples. An infrared camera was used to collect the data on the side, and the temperature field was analyzed by the relevant data analysis software, such as Infrec Analyzer, as shown in Figure 10.

**Figure 11:**
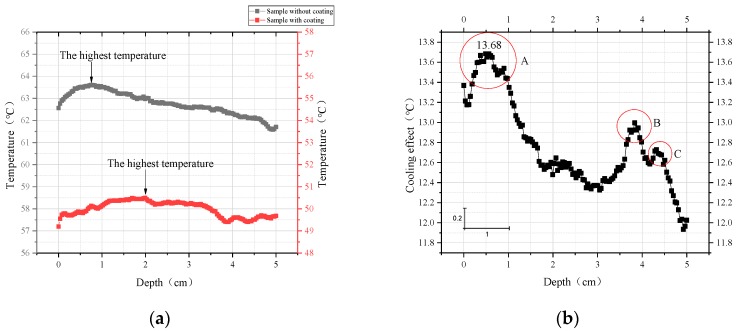
Analysis of internal temperature data. (**a**) Internal temperature changes of the coated and uncoated samples; (**b**) cooling effect of the TLCC.

Figure 11 illustrates that the coating significantly changed the temperature field inside the sample. Specifically, the cool coating had a cooling effect of more than 12 °C within 5 cm below the surface. In addition, we still found that the change of the temperature field had a certain regularity. First, Figure 11a shows that the highest temperature point inside the sample was not at the surface but at a certain distance below the surface, regardless of whether the coating was applied. The cool coating changed the temperature and depth of the hottest point. The cool coating not only reduced the maximum temperature of the asphalt concrete by 13.12 °C but also deepened the corresponding position by 1.24 cm. This means that the cool coating was important for relieving the high-temperature softening problem of asphalt pavements in summer. Second, the cooling effect in Figure 11b shows the temperature difference (Δt) between the coated and uncoated samples at the same position. It is not difficult to see that the cooling effect of the coating increased first with increasing depth, followed by a large peak (13.68 °C). Subsequently, the cooling effect was gradually reduced. However, the cooling effect suddenly appeared with two peaks at approximately 4 cm, which was due to the unevenness caused by the large pores inside the sample. However, this accidental error did not change the tendency of the cooling effect to gradually decrease. In general, the cool coating had a good cooling effect within 5 cm below the surface of the asphalt concrete pavement, and this effect showed a tendency to decrease slightly after the increase.

## 5. Conclusions and Outlook

In this paper, a novel cool coating (TLCC) was prepared that comprises three different structural layers: a reflecting layer, an emitting layer, and a thermal insulation layer. Then, we independently designed a CEED (cooling effect evaluation device) and a corresponding method for coatings, which were used to investigate the effect of each structural layer on the cooling effect and determine the content of various materials. Finally, we investigated the effect of this novel coating on the internal temperature field of asphalt concrete samples and studied the cooling effect of the coating inside the sample. According to the analysis of this article, the following results are obtained:

- We developed a CEED that can regulate various environmental parameters, including atmospheric temperature, humidity, and radiation intensity. This device improves the accuracy of the test results. At the same time, we proposed a corresponding design method, which includes the following four steps: Setting the environmental parameters, such as air temperature; checking the environmental parameters using a weather station; testing the cooling effect of the coating using temperature recorders and an infrared camera; and evaluating the cooling effect of the coating. This device and method take into account the environmental factors that influence the cooling effect of the coating and provide a reference for the accurate testing of the coating.
- Using the above device, we investigated the effects of different structural layers in the coating. The reflective layer is the main factor producing the coating cooling effect. On this basis, the addition of the emissive layer and the thermal insulation layer can improve the cooling effect of the coating. The reflective coating is capable of cooling the sample surface by 8.18 °C compared to the uncoated sample. The addition of the emissive layer can increase the cooling effect of the coating to 11.1 °C. The bottom thermal insulation layer can prevent heat from passing downward, which also increases the cooling effect to 13.25 °C. This information provides a reference to further optimize the structure of the coating.
- In the above coating structure, the contents of key materials are determined. The contents of titanium dioxide, carbon black, silicon dioxide, and hollow glass microspheres are 13.5%, 0.6%, 18%, and 17.5%, respectively.
- We analyzed the temperature field inside the samples with and without the cool coating (TLCC). We drew the following three conclusions: First, the cooling effect exceeds 12 °C regardless of any depth of the asphalt mixture sample (thickness of 5 cm); second, TLCC can reduce the maximum temperature inside the asphalt pavement (13.12 °C) and lower the maximum temperature’s corresponding position (1.24 cm); third, the cooling effect shows a tendency to increase first and then decrease slowly with an increase in depth. In general, this cool coating of the TLCC significantly changes the temperature field of the asphalt pavement.

This paper provides a reference for the structural optimization of the coating and a better cooling effect. At the same time, this study provides a more accurate and effective technical means for evaluating the cooling effect of the coating compared with other techniques. This study contributes to the reduction of road distresses, such as ruts, and the development of sustainable asphalt pavement. For future research, we propose the following suggestions:

- First, modify materials or find alternative new nanomaterials to develop high-performance coatings.
- Second, carry out finite element simulations and establish the temperature field inside the pavement to study the influence of relevant factors on the cooling effect of the coating, including solar radiation, temperature, and humidity.
- Third, it is necessary to establish a temperature field prediction model for asphalt pavement with a paved coating; the current temperature field prediction model is based on atmospheric temperature and solar radiation and does not consider whether the asphalt pavement surface is coated.

## Figures and Tables

**Table 1:** Technical characteristics of titanium oxide.

Appearance	Refractive Index	Average Particle Diameter	Brightness	pH	Density
White	2.8	0.20–0.26 µm	≥ 94.5%	6–9	4.23 g/cc

**Table 2:** Technical characteristics of silicon dioxide.

Appearance	Refractive Index	Average Particle Diameter	Boiling Point	pH	Density
White	1.553	8 µm	2950 °C	6.5–7.5	2.65 g/cc

**Table 3:** Technical characteristics of hollow glass microbeads.

Appearance	Density	Particle Size	Thermal Conductivity	pH
White	0.60 g/cc	15–120 µm	0.003–0.01 BTU/in⋅hr °F	8–9.5

**Table 4:** Coating scheme on test samples.

Scheme	Structures	Materials
Scheme 1	Reflective layer	Rutile titanium dioxide + Carbon black
Scheme 2	Reflective layer	Rutile titanium dioxide + Carbon black
	Emissive layer	Silicon dioxide
Scheme 3	Reflective layer	Rutile titanium dioxide + Carbon black
	Emissive layer	Silicon dioxide
	Thermal insulation layer	Hollow glass microbead
